# Associations between air pollution, accelerated biological aging, and oral inflammatory diseases: evidence from the UK Biobank cohort

**DOI:** 10.3389/fpubh.2026.1869182

**Published:** 2026-10-27

**Authors:** Hui Zhan, Dong Liu, You Guo, Wei Liu

**Affiliations:** 1 Department of Dentistry, The Central Hospital of Wuhan, Tongji Medical College, Huazhong University of Science and Technology, Wuhan, China; 2 Medical Big Data and Bioinformatics Research Centre, First Affiliated Hospital of Gannan Medical University, Ganzhou, China; 3 Ganzhou Key Laboratory of Environment and Health, Gannan Medical University, Ganzhou, Jiangxi, China; 4 Gannan Subcenter for Medical Genomics, National Genomics Data Center, Ganzhou, Jiangxi, China

**Keywords:** air pollution, association, biological aging, mediation analysis, oral inflammatory diseases

## Abstract

**Background:**

The relationships between air pollution exposure and incident risk of oral inflammatory diseases (OIDs) and roles of accelerated biological aging remain unclear.

**Methods:**

In 348,247 adults from the UK Biobank, the individual levels of air pollutants, including particulate matter (PM) (PM_2.5_, PM_10_, PM_2.5–10,_ and PM_2.5 absorbance_), nitrogen dioxide (NO_2_), nitric oxides (NO_x_), and air pollution score (multiple air pollutants exposure), were estimated. The associations between air pollution exposure and incident risk of OIDs were assessed. The mediation roles of the Klemera–Doubal method biological age (KDM-BA) acceleration and the phenotypic age (PhenoAge) acceleration were evaluated.

**Results:**

During the median follow-up of 16.49 years, a total of 3,967 incident OIDs were documented. Each one standard deviation increment in PM_2.5_, PM_10_, PM_2.5–10,_ PM_2.5 absorbance_, NO_2_, NO_x_, and air pollution score was significantly associated with 8%, 4%, 3%, 5%, 10%, 8%, and 8% increments in incident risk of OIDs, respectively (all *p* < 0.05). All air pollutants except PM_2.5–10_ were significantly and positively associated with KDM-BA acceleration, and all air pollutants were significantly and positively associated with PhenoAge acceleration (all *p* < 0.05). KDM-BA acceleration mediated the associations between all air pollution (except PM_2.5–10_) and incident risk of OIDs, with mediation proportion ranging from 1.21 to 5.42%. PhenoAge acceleration mediated the associations between all air pollutants and incident risk of OIDs, with mediation proportion ranging from 8.52 to 16.65%.

**Conclusion:**

Air pollution may contribute to increased incident risk of OIDs, and premature aging may play an important role in the pathogenic process.

## Introduction

OIDs are a group of common disorders of the oral mucosa, periodontal tissues, and glands caused by microbial infections, immune abnormalities, or physical and chemical stimuli. OIDs not only directly affect basic physiological functions including chewing and speech but also have a close association with multiple diseases, such as cardiovascular diseases, cancer, and mental disorders (1–3). Common OIDs include periodontitis, gingivitis, and oral mucositis. Their occurrence and development are the result of the combined effects of various factors such as genetic susceptibility, behavioral lifestyle, and environmental exposure. In recent years, with the acceleration of industrialization, air pollution has become a major public health issue of global concern. A large number of studies have confirmed that exposure to pollutants, such as PM and NO_2_, can significantly increase the risk of diseases of respiratory system, cardiovascular system, and nervous system (4–6). However, the association between air pollution and the health of the oral cavity directly connected to the external environment, especially the epidemiological evidence of its relationship with OIDs, is still relatively limited, and the conclusions are not very clear. The mouth serves as the gateway of the respiratory system and digestive system and is directly exposed to polluted air. Pollutants may affect the local immune microenvironment of the mouth through direct contact with mucosal tissues or indirectly through systemic inflammatory responses, thereby participating in the occurrence of oral diseases (7–9).

Aging is a complex biological process in which the functions of the body gradually decline over time. However, chronological age often does not fully reflect an individual’s true physiological condition. Therefore, scientists have proposed the concept of “accelerated biological aging”, which refers to the “premature aging” state where biological age exceeds chronological age (10). This accelerated aging state can be quantified through new biomarkers such as accelerated biological aging (11, 12). Accelerated biological aging can more sensitively capture the cumulative effect of environmental stress on the physiological wear of the body (13, 14). Air pollution has been proven to be an important environmental stressor that accelerates biological aging (15, 16). Air pollutants can drive cellular aging and tissue function deterioration through various molecular mechanisms such as inducing oxidative stress, triggering chronic low-grade inflammation, and causing telomere shortening, thereby increasing an individual’s biological age (17, 18). In addition, aging is an important risk factor for various oral diseases, especially chronic inflammatory diseases such as periodontitis (19, 20). Aging is closely related to the decline in host defense functions and the weakening of tissue repair capabilities associated with immunosenescence (19). Based on the existing evidence, a reasonable scientific hypothesis may be proposed: Air pollution exposure may be associated with increased risk of OIDs, and accelerated biological aging may play a key mediating role. In other words, air pollution may first prompt an individual’s biological age to accelerate through systemic pathways. This accelerated aging state may increase susceptibility to OIDs. Verifying this mediating hypothesis is expected to reveal a potential new pathway by which environmental pollution affects oral health.

Using data from the UK Biobank, a large prospective cohort, we estimated individual exposures to a panel of pollutants comprising PM (PM_2.5_, PM_10_, PM_2.5–10_, and PM_2.5 absorbance_), NO_2_, NO_x_, and air pollution scores combining multiple pollutants, derived from a high-resolution estimation model. The primary analysis focused on the association of these exposures with the risk of incident OIDs. Subsequently, accelerated biological aging was quantified. A formal mediation analysis framework was applied to elucidate the mediating role of this acceleration in the air pollution-OIDs pathway. These analyses aimed to provide a large-scale empirical basis for the hypothesis of “air pollution–biological aging–increased risk of OIDs”, thereby serving as a reference for the formulation of targeted public health and environmental policies.

## Methods

### Study population

In the UK Biobank, a prospective nationwide cohort study, the recruitment process involved the distribution of invitations to 9 million adults aged 40 and above between 2006 and 2010. A final cohort of more than half a million individuals was recruited, corresponding to a 5% response rate. The study protocol required attendance at one of 22 national assessment centers. The data collection process involved the completion of a touchscreen questionnaire, participation in a nurse-led interview, the provision of biological samples, and undergoing physical examinations, as documented in detail in a prior publication (21). A total of 348,247 participants who completed follow-up, had complete data on exposure and covariates, and had no history of OID were included in the study. The flow chart of population screening is shown in Supplementary Figure S1.

### Exposure determination

Annual concentrations of NO_2_ were modeled for 4 consecutive years (2005, 2006, 2007, and 2010). PM_10_ levels were estimated for 2007 and 2010, while other air pollutants were assessed for 2010 only. The 2010 annual average pollution levels were derived from a land use regression (LUR) model developed within the European Study of Cohorts for Air Pollution Effects (ESCAPE). These estimates were linked to participants’ geocoded residential addresses at the baseline visit. For the period 2005–2007, pollution data came from EU-wide air pollution maps with a 100 × 100 m resolution, which covered all participant residences. Further details regarding the LUR model and its performance are available elsewhere. In brief, leave-one-out cross-validation indicated good model performance (R-squared ranged from 68 to 89%). Following previous studies (2), we averaged the values across relevant years to represent long-term air pollution exposure (22). Drawing on previous studies, air pollution score was derived by adding up the concentrations of air pollutants which were weighted by the estimated risk of OID occurrence in this analysis (23, 24). Details of air pollution determination and air pollution score calculation are presented in Supplementary Methods.

### Outcome ascertainment

The incidence of OIDs was identified by using linkage with hospital admission data as of August 2025. OID events that first occurred during the follow-up period were identified. The detailed ICD-10 codes used to search OIDs events are shown in Supplementary Table S1.

### Covariates

The selection of covariates was based on a comprehensive consideration of the pre-analysis results, previous studies related to the exposure or outcome, and biological rationality (24–26). Covariates included age, gender, body mass index, ethnicity, Townsend deprivation index (an indicator of socioeconomic status, which incorporates unemployment, non-car ownership, non-home ownership, and household overcrowding), educational level (higher or equal to college/university level versus lower than college/university level), smoking history, alcohol consumption, and physical activity (active: engaging in moderate-intensity activities for at least 10 min each time for a total of five times per week, or undertaking high-intensity activities for at least 10 min each time for a total of three times per week; inactive: other).

### Accelerated biological aging

The KDM-BA and PhenoAge, two validated and widely used clinical parameter algorithms for assessing biological aging, were simultaneously constructed. In brief, KDM-BA is calculated through a regression analysis by integrating nine selected clinical biomarkers, namely, albumin, alkaline phosphatase, blood urea nitrogen, creatinine, C-reactive protein, forced expiratory volume in one second (FEV_1_), glycated hemoglobin, systolic blood pressure, and total cholesterol, with chronological age (11, 27). PhenoAge was derived from a mortality prediction score consisting of nine biomarkers, namely, albumin, alkaline phosphatase, creatinine, C-reactive protein, glucose, mean cell volume, red blood cell distribution width, white blood cell count, and lymphocyte ratio, and chronological age (28). The R software package *BioAge* was used to calculate the two biological ages (12). To reflect the rate of biological aging, the residuals of the two biological ages were calculated via natural spline regression method (29). Participants with a positive value for the rate of biological aging are classified as having a greater biological age, and vice versa (30). The selection of KDM-BA and PhenoAge as the primary mediating factors is based on several reasons: (i) they are derived from combinations of routine clinical biomarkers, ensuring high feasibility in large-scale epidemiological applications; (ii) their constituent biomarkers are mechanistically associated with systemic inflammation and physiological homeostasis—the key pathways through which air pollution is hypothesized to affect systemic and oral health; and (iii) they provide validated, comprehensive measures of organism-level physiological decline, making them suitable for capturing the cumulative, systemic biological aging process induced by long-term environmental stress.

### Statistical analyses

In descriptive analysis, categorical variables were exhibited as number (percentage), and continuous variables were exhibited as mean±standard deviation (SD) or median (first quartile and third quartile).

The associations between air pollution and incident risk of OIDs were evaluated by Cox proportional hazards model. Stratified analyses were conducted according to gender and age. Interaction effect was estimated by adding a product term of air pollution and stratified variable in the model (31). Sensitive analysis was conducted by classifying OIDs into two major subtypes: (1) periodontal diseases (such as periodontitis and gingivitis) and (2) oral mucosal and other inflammatory conditions (such as stomatitis, mucositis, and glossitis). The associations between air pollution and each subtype were examined separately.

General linear models were used to assess the association between air pollution and accelerated biological aging. Mediation analyses were conducted via R package *regmedint* (version 1.0.0). Mediation role of accelerated biological aging was analyzed by fitting the following two models (Equations 1, 2) (32, 33):

$$M=\theta_{1}+\beta_{\mathrm{air}\;\text{pollutant}}X_{\mathrm{air}\;\text{pollutant}}+\beta_{c}X_{c}+\lambda_{1}$$ (1)

$$Y=\theta_{2}+\mu_{\mathrm{air}\;\text{pollutant}}X_{\mathrm{air}\;\text{pollutant}}+\mathrm{\mu M}+\mu_{c}X_{c}+\lambda_{2}$$ (2)

Where *Y* represents incident risk of OIDs, *X_air pollutant_* represents the level of air pollution, *M* represents accelerated biological aging, *X_c_* represents covariate, *θ_1_* and *θ_2_* represent intercepts of the two models, *λ_1_* and *λ_2_* represent residuals of the two models, and *μ_air pollutant_* is direct effect of air pollution. Mediation effect is *β_air pollutant_*×*μ*, and mediation proportion is *β_air pollutant_*×*μ* /(*β_air pollutant_*×*μ + μ_air pollutant_*) × 100%. In the counterfactual framework implemented by the *regmedint* package, the outcome model for new-onset OID is indeed a Cox proportional hazards model. The confidence intervals for the direct effect, indirect effect, and mediating ratio are estimated using a non-parametric bootstrap method with 1,000 resamples.

All statistical analyses were performed via R software (version 4.1.0). A two-sided test with a *p*-value less than 0.05 was considered statistically significant.

## Results

### Basic characteristics of study population

The participant profile outlined in Table 1 indicates mean values of 56.2 years for age, 27.29 kg/m^2^ for body mass index, and −1.4 for the Townsend deprivation index. The sample consisted of 52.0% female, 95.5% white, 7.1% who were current smokers, 47.3% who consumed alcohol, and 53.8% who were physically active. Environmental exposure assessments revealed median concentrations of 9.9 μg/m^3^ for PM_2.5_, 38.2 μg/m^3^ for PM_10_, 6.1 μg/m^3^ for PM_2.5–10_, 1.1 × 10^−5^/m for PM_2.5 absorbance_, 27.8 μg/m^3^ for NO_2_, and 41.9 μg/m^3^ for NOx. The mean KDM-BA and PhenoAge values were close to the mean chronological age, at 56.2 and 56.1 years, respectively. A total of 3,967 incident OIDs were identified during the median follow-up duration of 16.49 years.

**Table 1:** Basic characteristics of the study population.

Characteristics	Total population
Number	348,247
Incident number of OIDs cases	3,967 (1.1)
Follow-up time, years	16.49 (15.8, 17.1)
Age, years	56.2 (8.1)
Female	181,174 (52.0)
Body mass index, kg/m^2^	27.29 (4.7)
Ethnicity	
White	332,660 (95.5)
Non-white	15,587 (4.5)
Educational level	
Higher or equal to college/university level	91,763 (26.34%)
Lower than college/university level	256,484 (73.66%)
Townsend deprivation index	−1.4 (2.9)
Smoking history	
Never	212,051 (60.9)
Former	111,439 (32.0)
Current	24,757 (7.1)
Alcohol consumption	
Drinker	164,789 (47.3)
Non-drinker	183,458 (52.7)
Active physical activity	187,356 (53.8)
Air pollution	
PM_2.5_, μg/m^3^	9.9 (9.2, 10.5)
PM_10_, μg/m^3^	38.2 (36.1, 40.7)
PM_2.5–10_, μg/m^3^	6.1 (5.8, 6.6)
PM_2.5 absorbance_, 10^5^/m^3^	1.1 (0.9, 1.3)
NO_2_, μg/m^3^	27.8 (22.7, 33.7)
NO_X_, μg/m^3^	41.9 (33.8, 50.5)
Air pollution score	−0.6 (−3.6, 2.8)
KDM-BA, years	56.2 (8.1)
KDM-BA acceleration, years	0 (9.6)
PhenoAge, years	56.1 (9.9)
PhenoAge acceleration, years	0 (5.3)

OIDs, oral inflammatory diseases; PM, particular matter; NO_2_, nitrogen dioxide; NO_X_, nitrogen oxides; KDM-BA, Klemera–Doubal method biological age; PhenoAge, phenotypic age.

### Associations between air pollution and incident risk of OIDs

As shown in Figure 1, all air pollutants, as well as air pollution score, were significantly and positively associated with incident risk of OIDs (all *p* < 0.05). Each one SD increment in PM_2.5_, PM_10_, PM_2.5–10,_ PM_2.5 absorbance_, NO_2_, NO_x_, and air pollution score was significantly associated with 8% (hazard ratio [HR]: 1.08; 95% confidence interval [CI]: 1.04, 1.11), 4% (HR: 1.04; 95% CI: 1.01, 1.07), 3% (HR: 1.03; 95% CI: 1.01, 1.07), 5% (HR: 1.05; 95% CI: 1.02, 1.08), 10% (HR: 1.10; 95% CI: 1.07, 1.13), 8% (HR: 1.08; 95% CI: 1.05, 1.12), and 8% (HR: 1.08; 95% CI: 1.05, 1.11) increments in incident risk of OIDs, respectively. When stratified according to gender or age, no significant interaction between gender or age and air pollution on incident risk of OIDs was observed (Supplementary Tables S2, S3). When stratifying according to disease type, the associations were broadly consistent across subtypes, suggesting the association between exposure and outcome is relatively stable (Supplementary Table S4).

**Figure 1:**
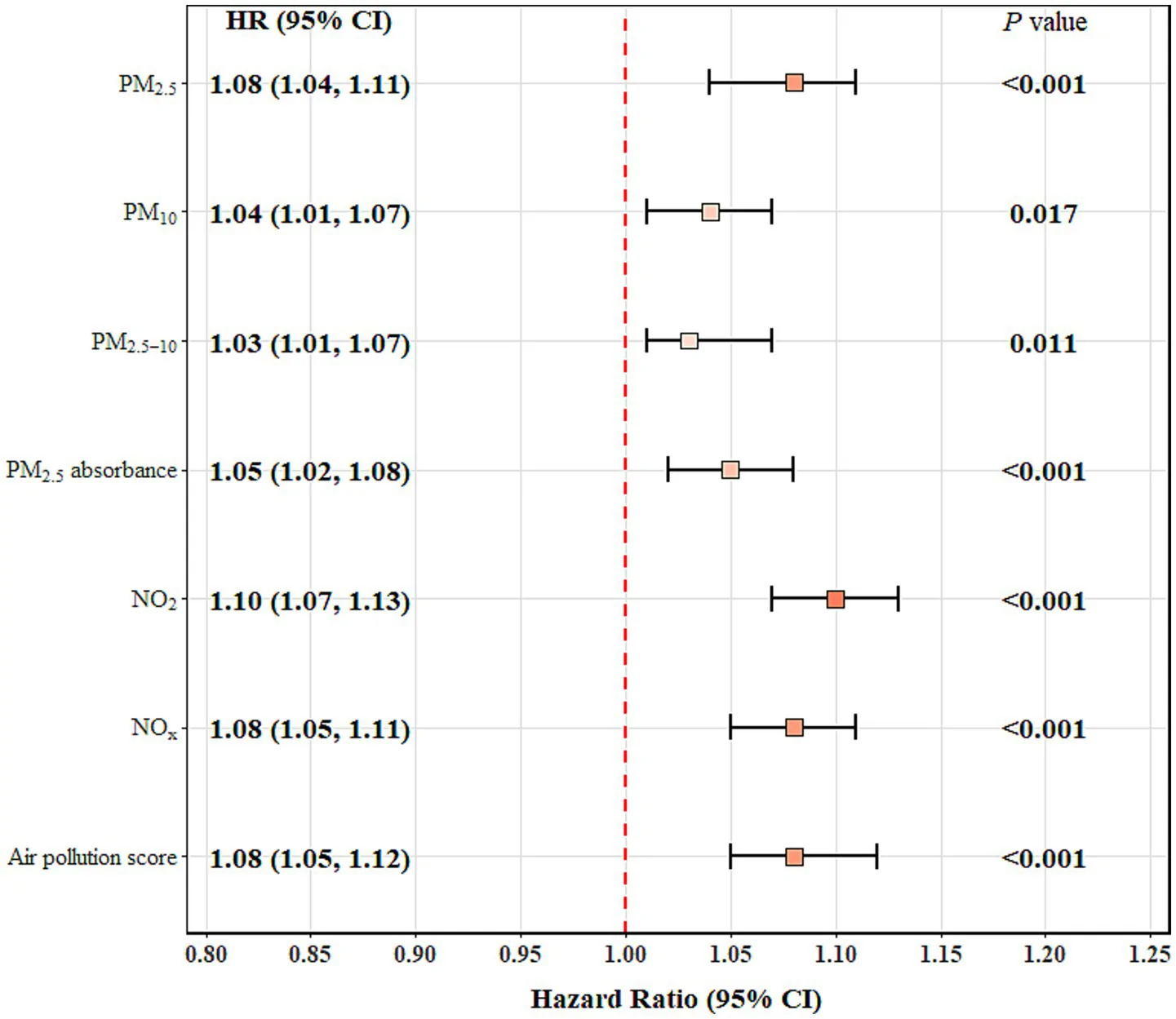
Associations between air pollution and the incident risk of OIDs in Cox proportional hazards models. OIDs, oral inflammatory diseases; PM, particular matter; NO_2_, nitrogen dioxide; NO_X_, nitrogen oxide; CI confidence interval. Models were adjusted for age, gender, body mass index, ethnicity, Townsend deprivation index, education level, smoking history, alcohol consumption, and physical activity.

### Mediation role of accelerated biological aging

The association between air pollution and accelerated biological aging is shown in Table 2. All air pollutants (except PM_2.5–10_) were significantly and positively associated with KDM-BA acceleration (all *p* < 0.05). Each one SD increment in PM_2.5_, PM_10_, PM_2.5 absorbance_, NO_2_, NO_x_, and air pollution score was significantly associated with 0.03 (95% CI: 0.01, 0.05), 0.02 (95% CI: 0.01, 0.03), 0.02 (95% CI: 0.01, 0.03), 0.02 (95% CI: 0.01, 0.04), 0.04 (95% CI: 0.02, 0.06), and 0.02 (95% CI: 0.01, 0.03) years increment in KDM-BA acceleration, respectively. All air pollutants were significantly and positively associated with PhenoAge acceleration (all *p* < 0.05). Each one SD increment in PM_2.5_, PM_10_, PM_2.5–10_, PM_2.5 absorbance_, NO_2_, NO_x_, and air pollution score was significantly associated with 0.10 (95% CI: 0.08, 0.11), 0.14 (95% CI: 0.12, 0.16), 0.05 (95% CI: 0.03, 0.07), 0.12 (95% CI: 0.10, 0.13), 0.14 (95% CI: 0.13, 0.16), 0.11 (95% CI: 0.09, 0.12), and 0.13 (95% CI: 0.12, 0.15) years increment in KDM-BA acceleration, respectively.

**Table 2:** Associations between air pollution and accelerated biological aging in linear regression.

Air pollution (each one standard deviation increment)	β (95% CI)	
	KDM-BA acceleration	PhenoAge acceleration
PM_2.5_, μg/m^3^	0.03 (0.01, 0.05)	0.10 (0.08, 0.11)
PM_10_, μg/m^3^	0.02 (0.01, 0.03)	0.14 (0.12, 0.16)
PM_2.5-10_, μg/m^3^	0.006 (−0.01, 0.01)	0.05 (0.03, 0.07)
PM_2.5 absorbance_, 10^5^/m^3^	0.02 (0.01, 0.03)	0.12 (0.10, 0.13)
NO_2_, μg/m^3^	0.02 (0.01, 0.04)	0.14 (0.13, 0.16)
NO_X_, μg/m^3^	0.04 (0.02, 0.06)	0.11 (0.09, 0.12)
Air pollution score	0.02 (0.01, 0.03)	0.13 (0.12, 0.15)

PM, particular matter; NO_2_, nitrogen dioxide; NO_X_, nitrogen oxides; KDM-BA, Klemera–Doubal method biological age; PhenoAge, phenotypic age; CI, confidence interval.

Models were adjusted for age, gender, body mass index, ethnicity, Townsend deprivation index, education level, smoking history, alcohol consumption, and physical activity.

As shown in Figure 2, KDM-BA acceleration significantly mediated the associations between all air pollutants (except PM_2.5–10_) and air pollution score and incident risk of OIDs, with mediation proportion ranging from 1.21 to 5.42%. PhenoAge acceleration significantly mediated the associations between all air pollutants and air pollution score and incident risk of OIDs, with mediation proportion ranging from 8.52 to 16.65%. The detail effect sizes are shown in Supplementary Tables S5, S6.

**Figure 2:**
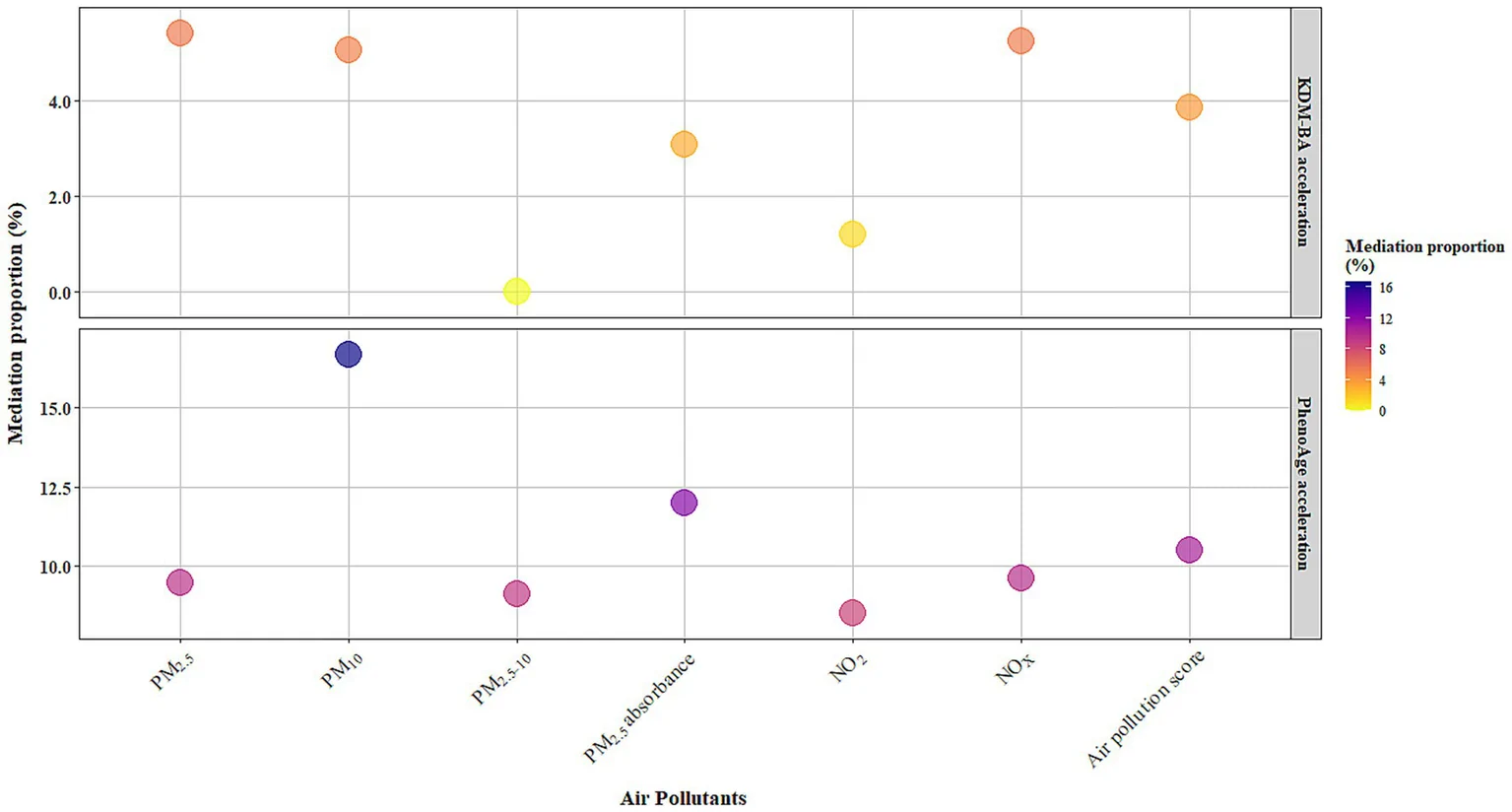
Mediation proportion of accelerated biological aging in associations between air pollution and incident risk of OIDs. OIDs, oral inflammatory diseases; PM, particular matter; NO_2_, nitrogen dioxide; NO_X_, nitrogen oxides; KDM-BA, Klemera–Doubal method biological age; PhenoAge, phenotypic age. Models were adjusted for age, gender, body mass index, ethnicity, Townsend deprivation index, education level, smoking history, alcohol consumption, physical activity, and accelerated biological aging.

## Discussion

This study, based on the large-scale prospective cohort of the UK Biobank, is the first to systematically explore the association between long-term air pollution exposure, accelerated biological aging, and the risk of new-onset OIDs at the national level. After a median follow-up of 16.49 years, our research results show that long-term exposure to air pollution is significantly associated with increased risk of OIDs. More importantly, two widely recognized accelerated biological aging indicators, namely, KDM-BA acceleration and PhenoAge acceleration, play a significant mediating role in this association. These findings suggest that incorporating biological aging markers into environmental health risk assessment systems and implementing oral health promotion strategies aimed at delaying biological aging for groups exposed to high-pollution environments may provide new and more forward-looking intervention ideas for the prevention of OIDs.

This study provides a strong supplement and extension to the existing evidence in the field of air pollution and oral health. Observational studies have revealed the association between air pollution and oral disease. For instance, a nationwide ecological study based on the US SEER database (2011–2019) found that each 1 μg/m^3^ increase in PM_2.5_ exposure level was associated with a 4% increment in risk of oral and pharyngeal cancer (34). In addition, a study published in 2024 based on data from the fourth national oral health survey in China indicated that long-term exposure to PM_2.5_ was significantly associated with the risk of periodontitis (35). Every 10 μg/m^3^ increase in the average concentration of PM_2.5_ was associated with increased risks of periodontitis of 9.0%, 8.0%, and 7.0% over 1, 3, and 5 years, respectively (35). The effect sizes observed in our study (a 3 to 10% increase in OID risk per standard deviation increment across various pollutants) are generally consistent in magnitude with these previous reports focusing on specific oral diseases. This concordance strengthens the plausibility of a genuine association between air pollution and a broader range of oral inflammatory conditions. However, most of these studies were cross-sectional designs or ecological studies, making it difficult to determine the temporal sequence and insufficient in assessing the combined effects of multiple pollutants. In contrast, this study is based on the large-scale prospective cohort design of the UK Biobank, with a median follow-up of 16.49 years. It not only evaluated the independent effects of multiple pollutants such as PM_2.5_, PM_10_, NO_2_, and NO_x_ but also adopted a comprehensive air pollution score, thereby more comprehensively reflecting the complex situation of mixed exposure to multiple pollutants in the real world. It is particularly worth noting that this study found that the risk associations between multiple pollutants and OIDs were all statistically significant. Although the effect sizes (a 3 to 10% increase in risk per SD increment) may seem small, given that air pollution is a widespread and difficult-to-avoid environmental exposure, the disease burden it causes at the population level could be quite significant. This characteristic of “low relative risk but high population risk” is consistent with the epidemiological pattern of air pollution’s impact on chronic diseases such as cardiovascular diseases, highlighting its importance in the field of public health.

The core finding of this study is the confirmation of the mediating role of accelerated biological aging in the association between air pollution and OIDs, which has solid biological rationality. Air pollution has been widely recognized as a strong inducer of oxidative stress and chronic low-grade inflammation (36). Air pollutants can enter the circulatory system through the respiratory tract or directly deposit on the oral mucosa, activating inflammatory signaling pathways, promoting the generation of reactive oxygen species, and leading to DNA damage, abnormal protein function, and mitochondrial dysfunction in cells, all of which are core drivers of cellular and overall organismal aging (10, 18). PhenoAge acceleration exhibited a stronger mediation proportion compared with KDM-BA acceleration. PhenoAge encompasses direct indicators of immune aging, such as lymphocyte percentage, as well as indicators of chronic inflammation/oxidative stress, such as red blood cell distribution width, which are key pathways activated by air pollution. In contrast, KDM-BA includes indicators that better reflect metabolism and organ function, such as blood urea nitrogen and forced expiratory volume. These findings suggest that air pollution may exert a more effective pathogenic impact on oral tissues by driving immune-inflammatory aging rather than merely through general metabolic decline (28). This finding highlights why PhenoAge may be a more sensitive and mechanism-consistent mediating factor in this specific exposure–disease context. The systemic inflammatory state induced by air pollution is precisely the key bridge connecting environmental pollution and local oral inflammation. The selection of KDM-BA and PhenoAge is particularly relevant for OIDs. The clinical biomarkers constituting these indices are not merely general aging markers but are mechanistically implicated in the pathogenesis of OIDs. For instance, systemic inflammation reflected by CRP can exacerbate local periodontal inflammation (37), and hypoalbuminemia can impair mucosal immunity and repair (38). Thus, an acceleration in these biological age scores reflects a deterioration of the systemic host environment that directly increases vulnerability to oral inflammatory breakdown, providing a plausible biological bridge between air pollution exposure and OID risk. However, the pattern for PM_2.5–10_, which showed a significant association with OID risk but a non-significant one with KDM-BA acceleration, is noteworthy and warrants comment, as it may reveal distinct pathogenic mechanisms. For instance, coarse particles may have a more pronounced local effect on the oropharyngeal mucosa, such as direct irritation and alteration of local microbiome, inducing the type of systemic physiological dysregulation quantified by KDM-BA (39). The significant mediation via PhenoAge for PM_2.5–10_ further supports the idea that inflammatory pathways remain relevant.

The significant direct impact of air pollutants on the risk of oral diseases persists even after considering the mediating role through biological aging, possibly reflecting the combined effect of these different pathways. These results indicate that air pollution affects oral health through a multifactorial model, in which accelerated systemic aging coexists with more direct local damage to the oral environment. For example, the direct effect of air pollutants on the oral mucosal epithelium can damage the epithelial physical barrier, regulate the composition of the oral microbiota, promote the growth of pathogenic bacteria, and trigger immune disorders through the “lung–mouth axis” or “stomach–mouth axis” (40–42). Accelerated biological aging is likely to be an important pathway that coexists and interweaves with these direct mechanisms. It represents the cumulative and systemic damage of environmental pollution to the physiological systems of the body, which reduces the ability of oral tissues to maintain homeostasis and resist infection, thereby increasing the risk of OID. The confirmation of this mediating pathway echoes recent findings in other disease areas (such as neurodegenerative diseases and thyroid dysfunction), which collectively emphasize that biological aging is a common pathway by which environmental stress leads to disease (15, 16).

The strengths of this study include a large-scale prospective design, long-term follow-up, assessment of multiple pollutants and a comprehensive pollution index, simultaneous use of two validated biological age algorithms, and rigorous statistical adjustment and mediation analysis. However, this study also has several limitations. First, although multiple known confounding factors have been controlled as much as possible, it is still impossible to completely rule out the possibility of residual confounding. For instance, poor oral hygiene habits are an important risk factor for oral diseases. Similarly, detailed occupational exposure situations or aspects of social and economic conditions beyond the Townsend deprivation index that have not been measured may be associated with higher pollution exposure and poorer health outcomes, potentially biasing the observed associations. Second, the assessment of air pollution exposure was based on model estimates of residential addresses rather than individual actual exposure monitoring, which may introduce measurement errors, but the method is widely recognized in large-scale epidemiological studies. Third, medical data might miss mild OID cases, and the findings of this study may mainly support the association between air pollution and the occurrence of OIDs requiring medical attention. Fourth, although the cohort size of the UK Biobank is large, the baseline response rate is only approximately 5%, and it is mainly composed of white, middle-aged, and older adults people from the UK, with overall health conditions superior to the national average (healthy volunteer bias). This limits the direct generalizability of our risk assessment results to other races, younger populations, and other geographical environments with different pollution combinations and baseline health conditions. Future validation is needed in more diverse cohorts. Fifth, our mediation analysis used the biological age acceleration calculated based on biomarkers measured only at the baseline. We cannot consider biological age or the changes in exposure over time, which may lead to an underestimation of the true mediating effect. Studies that repeat measurements of exposure and biomarkers will help to more accurately simulate these dynamic changes. Sixth, KDM-BA uses FEV_1_ as one of its nine component biomarkers. Since air pollution is a known risk factor for lung function decline, including FEV_1_ may lead to a certain degree of overlap between exposure and mediating factors, thereby potentially overestimating the mediating effect through the common respiratory pathway. However, after conducting a sensitivity analysis on KDM-BA without including FEV_1_, it was found that the results remained largely unchanged. Additionally, PhenoAge does not include FEV_1_ but shows a stronger and more consistent mediating effect, providing independent evidence for the biological aging pathway. Despite these limitations, the prospective design, control of key confounding factors, use of validated exposure models, and the strength and consistency of the observed associations all support the robustness of our main findings. Future studies can combine repeated measurements of biomarkers and individualized exposure data to validate our findings in different populations and further explore how air pollution affects oral health through more microscopic mechanisms such as epigenetic regulation.

## Conclusion

In conclusion, exposure to multiple environmental air pollutants is an independent risk factor for the onset of OIDs. More importantly, accelerated biological aging may act as a crucial mediator in the association between air pollution and OIDs. These findings have expanded our understanding of the impact of air pollution on oral health, going beyond the scope of local effects, and indicate that accelerating the overall biological aging process is a new pathway. From a public health perspective, this suggests that individuals living in severely polluted areas may benefit from monitoring indicators of biological aging. Moreover, intervention measures aimed at reducing air pollution and measures to prevent premature aging may have a synergistic effect in preventing OIDs. It is advocated that comprehensive strategies should be adopted in environmental and oral health policies.

## Data Availability

Publicly available datasets were analyzed in this study. This data can be found at: https://biobank.ndph.ox.ac.uk/.
