# Supplementary material for: Associations between air pollution, accelerated biological aging, and oral inflammatory diseases: evidence from the UK Biobank cohort
Source: Front Public Health. 2026 Oct 27;14:1869182. doi: 10.3389/fpubh.2026.1869182 (PMC13749453; doi:10.3389/fpubh.2026.1869182)
Supplement: Supplementary file 1 [file Table_2.docx]

**Supplementary Methods**

Annual concentration levels of NO_2_ were estimated in 4 consecutive years (2005, 2006, 2007, and 2010), whereas PM_10_ concentration levels were estimated in 2 years (2007 and 2010) and the other air pollutants levels were estimated in the year 2010. The annual average air pollution level in 2010 was estimated using a land use regression model developed as a part of the European Study of Cohorts for Air Pollution Effects (ESCAPE) and linked to participants’ geocoded residential addresses at the baseline visit [1]. In the UK Biobank, residential addresses were geocoded using the Ordnance Survey Postcode Address File, with a linkage rate of more than 95% for the full cohort [1]. Air pollution estimates during 2005–2007 were derived from EU-wide air pollution maps (resolution 100×100m), which covered the residential locations of the participants. More detailed information about the model and model performance has been described elsewhere [1]. In brief, leave-one-out cross-validation showed a good model performance (R-squared ranged from 68% to 89%). We averaged the values in different years to represent the long-term air pollution exposure in accordance with published studies [2]. We derived a air pollution composite metric through *β*-coefficient integration, expressed as: $\frac{6}{\Sigma\beta_{k}}\Sigma_{k=1}^{6}(\beta_{k}Pollutant$*_k_*$)$. where *β*-values represent ln-transformed hazard ratios quantifying pollutant-specific disease risks (Table S2). This *β*-weighting methodology follows established exposure aggregation frameworks [3,4].

**Reference:**

1.	Eeftens M, Beelen R, de Hoogh K, et al. Development of Land Use Regression models for PM(2.5), PM(2.5) absorbance, PM(10) and PM(coarse) in 20 European study areas; results of the ESCAPE project. *Environmental science & technology*. 2012;46(20):11195-11205.

2.	Zhang S, Qian ZM, Chen L, et al. Exposure to Air Pollution during Pre-Hypertension and Subsequent Hypertension, Cardiovascular Disease, and Death: A Trajectory Analysis of the UK Biobank Cohort. Environ Health Perspect. 2023;131(1):17008.

3. Wang M, Zhou T, Song Y, Li X, Ma H, Hu Y, et al. Joint exposure to various ambient air pollutants and incident heart failure: a prospective analysis in UK Biobank. *Eur Heart J*. 2021;42(16):1582-91.

4. Zhou L, Zhang Q, Luo H, Yu K, Meng X, Chen R, et al. Long-term exposure to air pollution and incidence risk of various arrhythmias: A prospective cohort study. Eco Environ Health. 2024;3(4):445-51.

Table S2. Associations between air pollution and the incident risk of OIDs according to gender in Cox proportional hazards models

| Air pollution (each one standard deviation-increment) | HR (95% CI) | | *P* for interaction |
| --- | --- | --- | --- |
|  | Male | Female |  |
| PM_2.5_, μg/m^3^ | 1.09 (1.04, 1.15) | 1.06 (1.02, 1.11) | 0.434 |
| PM_10_, μg/m^3^ | 1.04 (0.99, 1.09) | 1.04 (0.99, 1.08) | 0.792 |
| PM_2.5-10_, μg/m^3^ | 1.02 (0.97, 1.07) | 1.05 (1.01, 1.09) | 0.329 |
| PM_2.5 absorbance_, 10 ^5^/m^3^ | 1.06 (1.02, 1.11) | 1.04 (1.001, 1.08) | 0.341 |
| NO_2_, μg/m^3^ | 1.10 (1.05, 1.15) | 1.09 (1.05, 1.14) | 0.635 |
| NO_X_, μg/m^3^ | 1.10 (1.05, 1.15) | 1.08 (1.05, 1.11) | 0.323 |
| Air pollution score | 1.09 (1.04, 1.14) | 1.07 (1.03, 1.12) | 0.597 |

Abbreviations: OIDs, oral inflammatory diseases; PM, particular matter; NO_2_, nitrogen dioxide; NO_X_, nitrogen oxide; HR, hazard ratio; CI, confidence interval.

Models were adjusted for age, gender, body mass index, ethnicity, Townsend deprivation index, education level, smoking status, drinking status, and physical activity.

*P* for interaction was estimated by adding product term of exposure and stratified variable in the model.

Table S3. Associations between air pollution and the incident risk of OIDs according to age in Cox proportional hazards models

| Air pollution (each one standard deviation-increment) | HR (95% CI) | | *P* for interaction |
| --- | --- | --- | --- |
|  | Age<55 years | Age≥55 years |  |
| PM_2.5_, μg/m^3^ | 1.08 (1.03, 1.13) | 1.08 (1.03, 1.12) | 0.798 |
| PM_10_, μg/m^3^ | 1.02 (0.97, 1.07) | 1.05 (1.01, 1.09) | 0.377 |
| PM_2.5-10_, μg/m^3^ | 1.03 (0.98, 1.08) | 1.04 (1.01, 1.08) | 0.471 |
| PM_2.5 absorbance_, 10 ^5^/m^3^ | 1.05 (1.01, 1.11) | 1.05 (1.01, 1.09) | 0.683 |
| NO_2_, μg/m^3^ | 1.10 (1.07, 1.13) | 1.11 (1.06, 1.15) | 0.542 |
| NO_X_, μg/m^3^ | 1.08 (1.03, 1.14) | 1.08 (1.04, 1.12) | 0.873 |
| Air pollution score | 1.07 (1.02, 1.13) | 1.09 (1.04, 1.13) | 0.785 |

Abbreviations: OIDs, oral inflammatory diseases; PM, particular matter; NO_2_, nitrogen dioxide; NO_X_, nitrogen oxide; HR, hazard ratio; CI, confidence interval.

Models were adjusted for age, gender, body mass index, ethnicity, Townsend deprivation index, education level, smoking status, drinking status, and physical activity.

*P* for interaction was estimated by adding product term of exposure and stratified variable in the model.

Table S4. Associations between air pollution and the incident risk of OIDs according to disease type in Cox proportional hazards models

| Air pollution (each one standard deviation-increment) | HR (95% CI) | |
| --- | --- | --- |
|  | Periodontal diseases | Oral mucosal and other inflammatory conditions |
| PM_2.5_, μg/m^3^ | 1.09 (1.01, 1.12) | 1.08 (1.02, 1.11) |
| PM_10_, μg/m^3^ | 1.05 (1.01, 1.11) | 1.05 (1.002, 1.10) |
| PM_2.5-10_, μg/m^3^ | 1.04 (0.99, 1.09) | 1.06 (1.02, 1.10) |
| PM_2.5 absorbance_, 10 ^5^/m^3^ | 1.06 (1.01, 1.11) | 1.05 (1.01, 1.10) |
| NO_2_, μg/m^3^ | 1.09 (1.05, 1.12) | 1.12 (1.07, 1.17) |
| NO_X_, μg/m^3^ | 1.09 (1.04, 1.15) | 1.07 (1.05, 1.14) |
| Air pollution score | 1.08 (1.03, 1.15) | 1.10 (1.05, 1.15) |

Abbreviations: OIDs, oral inflammatory diseases; PM, particular matter; NO_2_, nitrogen dioxide; NO_X_, nitrogen oxide; HR, hazard ratio; CI, confidence interval.

Models were adjusted for age, gender, body mass index, ethnicity, Townsend deprivation index, education level, smoking status, drinking status, and physical activity.

Table S5. Mediation analysis of KDM-BA acceleration in associations between air pollution and the incident risk of OIDs

| Air pollution (each one standard deviation-increment) | Total effect (95% CI) | Direct effect (95% CI) | Indirect effect  (95% CI) | Mediation proportion |
| --- | --- | --- | --- | --- |
| PM_2.5_, μg/m^3^ | 1.10 (1.07, 1.13) | 1.09 (1.06, 1.13) | 1.004 (1.003, 1.006) | 5.42% |
| PM_10_, μg/m^3^ | 1.05 (1.01, 1.09) | 1.04 (1.01, 1.08) | 1.002 (1.001, 1.003) | 5.07% |
| PM_2.5-10_, μg/m^3^ | 1.04 (1.01, 1.08) | 1.04 (1.01, 1.08) | 1.002 (0.999, 1.008) | - |
| PM_2.5 absorbance_, 10 ^5^/m^3^ | 1.08 (1.04, 1.11) | 1.07 (1.04, 1.12) | 1.002 (1.001, 1.003) | 3.08% |
| NO_2_, μg/m^3^ | 1.11 (1.07, 1.15) | 1.10 (1.06, 1.15) | 1.002 (1.001, 1.003) | 1.21% |
| NO_X_, μg/m^3^ | 1.11 (1.06, 1.14) | 1.10 (1.06, 1.13) | 1.004 (1.003, 1.005) | 5.26% |
| Air pollution score | 1.11 (1.07, 1.14) | 1.10 (1.06. 1.13) | 1.003 (1.002, 1.004) | 3.87% |

Abbreviations: OIDs, oral inflammatory diseases; PM, particular matter; NO_2_, nitrogen dioxide; NO_X_, nitrogen oxides; KDM-BA, Klemera-Doubal method biological age; CI, confidence interval.

Models were adjusted for age, gender, body mass index, ethnicity, Townsend deprivation index, education level, smoking status, drinking status, physical activity, and accelerated biological aging.

Table S6. Mediation analysis of PhenoAge acceleration in associations between air pollution and the incident risk of OIDs

| Air pollution (each one standard deviation-increment) | Total effect  (95% CI) | Direct effect (95% CI) | Indirect effect  (95% CI) | Mediation proportion |
| --- | --- | --- | --- | --- |
| PM_2.5_, μg/m^3^ | 1.10 (1.06, 1.13) | 1.09 (1.05, 1.12) | 1.007 (1.006, 1.009) | 9.49% |
| PM_10_, μg/m^3^ | 1.05 (1.02, 1.08) | 1.04 (1.01, 1.08) | 1.008 (1.007, 1.010) | 16.65% |
| PM_2.5-10_, μg/m^3^ | 1.04 (1.01, 1.07) | 1.04 (1.01, 1.07) | 1.003 (1.002, 1.004) | 9.13% |
| PM_2.5 absorbance_, 10 ^5^/m^3^ | 1.06 (1.03, 1.10) | 1.06 (1.02, 1.09) | 1.007 (1.006, 1.009) | 12.01% |
| NO_2_, μg/m^3^ | 1.11 (1.08, 1.14) | 1.10 (1.07, 1.13) | 1.008 (1.007, 1.010) | 8.52% |
| NO_X_, μg/m^3^ | 1.10 (1.06, 1.13) | 1.09 (1.06, 1.12) | 1.008 (1.007, 1.010) | 9.64% |
| Air pollution score | 1.10 (1.07. 1.15) | 1.09 (1.06, 1.12) | 1.008 (1.006, 1.009) | 10.51% |

Abbreviations: OIDs, oral inflammatory diseases; PM, particular matter; NO_2_, nitrogen dioxide; NO_X_, nitrogen oxides; PhenoAge, phenotypic age; CI, confidence interval.

Models were adjusted for age, gender, body mass index, ethnicity, Townsend deprivation index, education level, smoking status, drinking status, physical activity, and accelerated biological aging.

Figure 1. Flow chart of study population screening.
